# Low Socioeconomic Status at the Patient and Institutional Level Impact Achievement of Breast Cancer Quality Standards

**DOI:** 10.1245/s10434-025-17529-w

**Published:** 2025-06-02

**Authors:** Kristen M. HoSang, Richard J. Bleicher, Katharine A. Yao, Jill R. Dietz, Austin D. Williams

**Affiliations:** 1 Department of Surgery, Temple University Hospital, Philadelphia, PA USA; 2 Department of Surgical Oncology, Fox Chase Cancer Center, Philadelphia, PA USA; 3 Department of Surgery, NorthShore University Health System, Evanston, IL USA; 4 American Society of Breast Surgeons, Columbia, MD USA; 5 The Data Working Group, National Accreditation Program for Breast Centers, American College of Surgeons, Chicago, IL USA

**Keywords:** Breast cancer, Socioeconomic status, Quality measures, National accreditation program of breast centers

## Abstract

**Background:**

The National Accreditation Program of Breast Centers (NAPBC) developed comprehensive, multidisciplinary standards to improve quality outcomes. We have previously shown that institutions treating larger proportions of breast cancer patients of low socioeconomic status (SES) achieve these benchmarks at lower rates. This study assesses whether a patient’s SES interacts with an institution’s SES mix in achieving the 2018 NAPBC standards.

**Methods:**

Using the National Cancer Database, low SES patients were defined by insurance status, income and educational attainment, and low SES institutions were defined as the decile containing the largest proportion of low SES patients. Cohorts were created for each measurable NAPBC standard: breast-conserving surgery, sentinel lymphadenectomy, adjuvant radiation, adjuvant chemotherapy, and adjuvant endocrine therapy. Adjusted odds of standard-compliant treatment (SCT) were analyzed.

**Results:**

We included 3,156,604 patients (23% being low SES) treated at 1180 non-low SES institutions and 283,155 patients (59% being low SES) treated at 131 low SES institutions. All subgroups reached the 50% threshold for breast-conserving surgery, with rates of SCT > 70% for all other standards. Both low SES institution and low SES patient status were independently associated with a lower probability of SCT for all standards (*p* < 0.001), except there was no association between institution type and receiving adjuvant radiation (*p* = 0.21).

**Conclusions:**

Patient and institution SES impact the achievement of NAPBC standards; low SES patients received lower SCT regardless of institution SES. Further work to create strategies that address SES-related disparities are imperative to provide equitable care.

**Supplementary Information:**

The online version contains supplementary material available at 10.1245/s10434-025-17529-w.

Breast cancer is the most prevalent noncutaneous cancer in women with increasing global incidence.^1^ Despite improvements in screening, diagnosis, and treatments, it remains the second leading cause of cancer deaths in non-Black/non-Hispanic women and the leading cause in Black and Hispanic women.^2^ In addition to racial disparities, treatments and outcomes vary by socioeconomic status (SES). Lower SES women have lower screening mammography rates, more advanced disease at diagnosis, lower rates of standard-compliant treatment (SCT), and higher mortality rates than women of a higher SES.^3–5^ These disparities are driven by factors, such as tumor biology, access to quality care, and social or institutional barriers that impact care.

One way to assess these disparities is through national accreditation groups. The National Accreditation Program for Breast Centers (NAPBC) is a coalition of multiple professional organizations, sponsored by the American College of Surgeons, and is dedicated to improving the quality of care for breast cancer patients.^6^ Breast centers are accredited by the NAPBC by demonstrating compliance with 28 multidisciplinary standards that assess the quality of care provided by the center throughout a patient’s journey from screening to survivorship.^7^ In a previous study, we found significant differences in how institutions that care for higher proportions of low-SES patients meet these standards.^8^ These results raise concern that treatment differences between institution types may exacerbate disparities already faced by patients of a low SES.

To further understand the institutional impact on the quality of breast cancer care in the context of individual SES, we sought to investigate whether a patient’s SES has any association with an institution’s SES and ultimately how that impacts the receipt of multidisciplinary NAPBC standards.

## Methods

After institutional review board approval, we performed a retrospective analysis of the National Cancer Database (NCDB). This is a collaboration between the American College of Surgeons and the American Cancer Society, in which patient-level data are collected from cancer patients seen at Commission on Cancer (CoC) accredited programs representing approximately 70% of United States cancer cases.^9,10^ Of note, while all programs submitting data to the NCDB are CoC-accredited, approximately one-third are NAPBC-accredited (estimates given by American College of Surgeons Cancer Programs staff).

Female patients treated from 2004 to 2019 were identified from the NCDB breast cancer Participant User File (PUF) and were categorized as either non-low SES or low SES. Low SES was defined as meeting at least one of the following criteria: uninsured; on Medicaid; or in the lowest quartiles for income and educational attainment (the latter two being related to one’s area of residence, as personal income or education are not coded in the NCDB). We excluded patients for whom insurance status, income, and educational quartiles were all missing.

Next, we identified institutions that reported at least 100 breast cancer patients to the NCDB between 2004 and 2019. We ranked each institution based on the percent of patients who were low SES and made a composite ranking for each institution. For the purposes of this study. the 10% of institutions having the highest proportion of low SES patients were categorized as low SES institutions, whereas the other 90% were categorized as non-low SES institutions. We chose the 10% threshold to assess institutions treating patients at the most extreme margin of socioeconomic status while maintaining adequate power for comparisons between patients treated at each institution type. We compared patient demographics and tumor characteristics between low and nonlow SES patients stratified by low and nonlow SES institutions.

The 2018 NAPBC accreditation standards were reviewed, and we identified five standards and performance measures that are measurable using data in the NCDB (Table 1). Overall adherence to these standards (and others not measurable with NCDB data) is monitored by the NAPBC and the CoC for the purposes of accreditation. We then created analysis cohorts based on the specification for patient and tumor factors of the identified NAPBC standards. Each cohort was stratified by institution type then patient demographics, tumor characteristics, and the rates of meeting the specific standard were compared between low and nonlow SES patients. Given differences in patient and tumor characteristics and to determine the relative contribution of patient and institution factors, univariate and multivariate logistic regression models were made for each standard to calculate the adjusted odds of meeting the standard for both patient and institution SES status.

**Table 1:** Selected standards and performance measures from the 2018 National Accreditation Program for Breast Centers Standards Manual assessed in this study

Selected 2018 NAPB Standards and Performance Measures		
Standard Number	Abbreviation	Text from NAPBC Standards Manual
2.3	BCS	Breast-conserving surgery is offered to appropriate patients with breast cancer. A target rate of at least 50% of all eligible patients diagnosed with early stage breast cancer (Stage 0, I, II) is treated with breast conserving surgery.
2.4	SLNB	Axillary sentinel lymph node biopsy is considered or performed for patients with early-stage breast cancer (clinical Stage I, II)
2.12	XRT	Radiation therapy is administered within 1 year (365 days) of diagnosis for women under age 70 receiving breast-conserving surgery for breast cancer.
2.13	Endocrine	Combination chemotherapy is considered or administered within 4 months (120 days) of diagnosis for women under the age of 70 with AJCC T1c, Stage II, or Stage III hormone receptor-negative breast cancer.
2.13	Chemo	Tamoxifen or third-generation aromatase inhibitor is considered or administered within 1 year (365 days) of diagnosis for women with AJCC T1c, Stage II, or Stage III hormone-receptor-positive breast cancer.

*AJCC* American Joint Committee on Cancer

All statistical analyses were performed with SPSS statistical software (version 28.0, IBM Corp., Armonk, NY). Cases with missing data were excluded from analysis on an analysis-by-analysis basis. Comparisons of patient demographic data were performed by using independent *t*-tests, one-way ANOVA and chi-square tests. Binary logistic regression was used to calculate odds ratios in univariate and multivariable analyses of the likelihood of or differences in meeting NAPBC standards. All *p* values were derived from two-tailed tests with significance as *p* < 0.05.

## Results

Of the 1333 institutions reporting breast cancer cases to the NCDB, 22 were excluded from analysis for reporting fewer than 100 cases from 2004 to 2019. After performing composite ranking, 140 institutions (11%) were grouped as low SES institutions, leaving 1179 as nonlow SES institutions.

Overall, 3,439,759 female patients with breast cancer were reported by these 1311 institutions; 5185 patients did not meet inclusion criteria. Of the remaining patients, 899,686 (26.2%) were categorized as low SES, leaving 2,540,073 (73.8%) categorized as nonlow SES patients. Being insured by Medicaid (which is a patient-specific variable) qualified 203,315 patients (23%) as low SES and being uninsured qualified 64,294 patients (7%). The remainder (70%) qualified based on income and educational quartiles.

When stratified by institution and patient type, 23% of the 3,156,604 patients treated at nonlow SES institutions were low SES patients, whereas 59% of the 283,155 patients treated at low SES institutions were low SES patients. The patient demographics and tumor characteristics of each group are summarized in Table 1. Owing to the large cohort size, each comparison (both within and between institution type) were significant as *p* < 0.001. While this limits precise evaluation, clinically relevant differences include a higher proportion of non-White patients, higher clinical and pathologic stages, high tumor grade and triple negative breast cancers among low SES patients and those treated at low SES institutions.

### Breast-Conserving Surgery

**Fig. 1:**
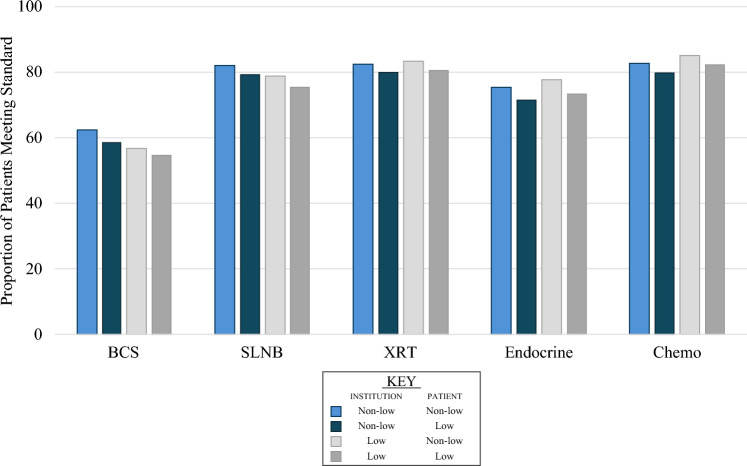
Unadjusted rates of meeting NAPBC quality standards stratified by patient and institution socioeconomic status. *BCS* breast-conserving surgery; *SLNB* sentinel lymphadenectomy; *XRT* adjuvant radiation; *Endocrine* adjuvant endocrine therapy; *Chemo* adjuvant chemotherapy

The cohort of patients with Stage 0, I, or II breast cancer meeting NAPBC criteria for consideration for breast-conserving surgery (BCS) was composed of 2.4 million patients, 180,116 (8%) of whom were treated at low SES institutions (Supplementary Table 1). As in the overall cohort, there were significant differences in patient demographics and tumor characteristics both within and between institution type (all *p* < 0.001). When stratified by institution and patient SES, all subgroups met the 50% quantitative NAPBC standard for BCS, which we measured using performance of BCS and without knowledge of whether the patients were indeed BCS-candidates (Fig. 1). Low SES institutions had a lower unadjusted BCS rate than nonlow SES institutions (56% vs. 62%, respectively, *p* < 0.001) and low SES patients had a lower unadjusted BCS rate than nonlow SES patients at both institution types (*p* < 0.001). The lowest unadjusted rate of BCS was among low SES patients treated at low SES institutions (55%), whereas the highest rate was among nonlow SES patients treated at nonlow SES institutions (63%) (Table 2).

**Table 2:** Demographic and clinicopathologic features of the source cohort without selections for specific NAPBC standards stratified by institution and patient socioeconomic status

	Nonlow SES institutions		Low SES institutions	
	Nonlow SES patients	Low SES patients	Nonlow SES patients	Low SES patients
*n*	2,423,051	733,553	117,022	166,133
Age	61.7± 13.2	59.9 ± 13.3	61.9 ± 12.9	59.5 ± 13.1
*Race*				
White	2,115,988 (87.3)	525,938 (71.7)	94,173 (80.5)	106,765 (64.3)
Black	171,363 (7.1)	151,743 (20.7)	18,809 (16.1)	51,696 (31.1)
Other	111,082 (4.6)	47,358 (6.5)	3,351 (2.9)	6,323 (3.8)
Unknown	24,618 (1)	8,514 (1.2)	689 (0.6)	1,349 (0.8)
*Insurance status*				
Not insured	0 (0)	42,481 (5.8)	0 (0)	21,798 (13.1)
Private insurance	1,399,822 (57.8)	266,702 (36.4)	61,279 (52.4)	50,156 (30.2)
Medicaid	0 (0)	170,019 (23.2)	0 (0)	33,223 (20)
Medicare	960,188 (39.6)	236,435 (32.2)	50,223 (42.9)	55,290 (33.3)
Other government	24,821 (1)	5,437 (0.7)	2,880 (2.5)	1,442 (0.9)
Unknown	38,220 (1.6)	12,479 (1.7)	2,640 (2.3)	4,224 (2.5)
*Percent no high school degree*				
17.6% or more	0 (0)	432,913 (59)	0 (0)	117,072 (70.5)
10.9–17.5%	488,176 (20.1)	167,993 (22.9)	41,863 (35.8)	33,016 (19.9)
6.3–10.8%	763,237 (31.5)	72,134 (9.8)	33,777 (28.9)	8,638 (5.2)
Less than 6.3%	839,455 (34.6)	35,966 (4.9)	24,446 (20.9)	3,021 (1.8)
Unknown	332,183 (13.7)	24,547 (3.3)	16,936 (14.5)	4,386 (2.6)
*Median income quartiles*				
Less than $40,227	0 (0)	358,279 (48.8)	0 (0)	111,933 (67.4)
$40,227–$50,353	381,302 (15.7)	153,633 (20.9)	37,037 (31.6)	30,724 (18.5)
$50,354–$63,332	539,343 (22.3)	117,388 (16)	33,963 (29)	12,652 (7.6)
$63,333 or more	1,167,534 (48.2)	78,395 (10.7)	28,815 (24.6)	5,975 (3.6)
Unknown	334,872 (13.8)	25,858 (3.5)	17,207 (14.7)	4,849 (2.9)
*Institution type*				
Community cancer center	157,326 (6.5)	52,937 (7.2)	6,921 (5.9)	20,076 (12.1)
Comprehensive community cancer program	983,255 (40.6)	299,486 (40.8)	39,767 (34)	48,767 (29.4)
Academic/research program	637,365 (26.3)	197,370 (26.9)	53,984 (46.1)	76,147 (45.8)
Integrated network cancer program	548,078 (22.6)	141,175 (19.2)	11,466 (9.8)	11,354 (6.8)
Unknown	97,027 (4)	42,585 (5.8)	4,884 (4.2)	9,789 (5.9)
*Charlson/Deyo score*				
0	2,060,642 (85)	599,737 (81.8)	97,051 (82.9)	134,150 (80.7)
1	278,292 (11.5)	100,454 (13.7)	15,224 (13)	24,274 (14.6)
2	58,525 (2.4)	22,892 (3.1)	3,228 (2.8)	5,185 (3.1)
≥ 3	25,592 (1.1)	10,470 (1.4)	1,519 (1.3)	2,524 (1.5)
*AJCC clinical stage*				
0	437,628 (18.1)	115,741 (15.8)	18,197 (15.6)	23,058 (13.9)
1	941,003 (38.8)	242,972 (33.1)	42,929 (36.7)	50,534 (30.4)
2	400,120 (16.5)	143,618 (19.6)	20,984 (17.9)	34,731 (20.9)
3	98,183 (4.1)	46,542 (6.3)	5,936 (5.1)	12,669 (7.6)
4	77,622 (3.2)	38,535 (5.3)	4,531 (3.9)	10,554 (6.4)
Unknown	468,495 (19.3)	146,145 (19.9)	24,445 (20.9)	34,587 (20.8)
*AJCC pathologic stage*				
0	388,445 (16)	103,714 (14.1)	18,007 (15.4)	22,782 (13.7)
1	971,578 (40.1)	251,345 (34.3)	45,509 (38.9)	53,427 (32.2)
2	442,402 (18.3)	149,284 (20.4)	22,397 (19.1)	35,435 (21.3)
3	132,377 (5.5)	53,281 (7.3)	7,435 (6.4)	14,100 (8.5)
4	36,986 (1.5)	16,986 (2.3)	2,059 (1.8)	4,136 (2.5)
Unknown	451,263 (18.6)	158,943 (21.7)	21,615 (18.5)	36,253 (21.8)
*Tumor subtype*				
HR+ HER2−	962,286 (39.7)	273,664 (37.3)	46,971 (40.1)	60,536 (36.4)
HER2+	182,636 (7.5)	64,714 (8.8)	10,295 (8.8)	16,461 (9.9)
HR− HER2−	138,501 (5.7)	54,942 (7.5)	8,553 (7.3)	15,064 (9.1)
Unknown	1,139,628 (47)	340,233 (46.4)	51,203 (43.8)	74,072 (44.6)
*Tumor grade*				
Well-differentiated	479,200 (19.8)	123,772 (16.9)	21,697 (18.5)	25,748 (15.5)
Moderately differentiated	943,014 (38.9)	270,337 (36.9)	43,603 (37.3)	58,355 (35.1)
Poorly differentiated	670,378 (27.7)	230,804 (31.5)	34,104 (29.1)	55,200 (33.2)
Unknown	330,459 (13.6)	108,640 (14.8)	17,618 (15.1)	26,830 (16.1)

All comparisons between and within institution SES categories are statistically significant with a *p* < 0.001

*NAPBC* National Accreditation Program of Breast Centers; *SES* socioeconomic status; *HR* hormone receptor

Both low institution and patient SES were associated with lower odds of BCS on univariate (odds ratio [OR] 0.78, *p* < 0.001; OR 0.84, *p* < 0.001, respectively) and multivariable analysis when adjusted for clinicopathologic features (OR 0.93, *p* < 0.001; OR 0.94, *p* < 0.001, respectively; Table 3). Interestingly, increasing age, Black race and TNBC were all associated with higher odds of BCS.

**Table 3:** Univariate and multivariable analyses of the odds of patients undergoing guideline concordant care according to breast conserving surgery, adjuvant radiation therapy, and sentinel lymphadenectomy standards

	Breast-conserving surgery						Adjuvant radiation						Sentinel lymphadenectomy					
	Univariate			Multivariate			Univariate			Multivariate			Univariate			Multivariate		
	OR	95% CI	*p*	OR	95% CI	*p*	OR	95% CI	*p*	OR	95% CI	*p*	OR	95% CI	*p*	OR	95% CI	*p*
Age	1.02	1.02–1.02	<0.001	1.02	1.02–1.02	<0.001	1.02	1.02–1.03	<0.001	1.01	0.99–1.02	0.39	0.96	0.96–0.96	<0.001	0.95	0.95–0.95	<0.001
*Race*																		
White (ref)																		
Black	0.91	0.900.92	<0.001	1.12	1.10–1.13	<0.001	0.33	0.29–0.38	<0.001	0.54	0.44–0.66	<0.001	0.83	0.82–0.84	<0.001	0.84	0.82–0.85	<0.001
Other	0.79	0.78–0.80	<0.001	0.89	0.87–0.90	<0.001	0.78	0.59–1.02	0.07	1.09	0.73–1.64	0.67	1.08	1.06–1.11	<0.001	0.95	0.93–0.98	<0.001
*Institution type*																		
Community cancer center (ref)																		
Comprehensive community cancer program	0.93	0.92–0.94	<0.001	0.90	0.89–0.91	<0.001	1.36	1.07–1.73	0.01	1.27	0.93–1.74	0.13	1.27	1.25–1.30	<0.001	1.21	1.19–1.23	<0.001
Academic/research program	0.88	0.87–0.89	<0.001	0.92	0.91–0.94	<0.001	1.02	0.80–1.29	0.90	1.20	0.88–1.64	0.26	1.27	1.25–1.30	<0.001	1.18	1.16–1.21	<0.001
Integrated network cancer program	0.91	0.90 0.93	<0.001	0.89	0.88–0.91	<0.001	1.57	1.21–2.05	<0.001	1.48	1.05–2.10	0.03	1.50	1.47–1.53	<0.001	1.41	1.38–1.44	<0.001
*Charlson/Deyo score*																		
0 (ref)																		
1	0.96	0.95–0.97	<0.001	0.91	0.91–0.92	<0.001	0.71	0.60–0.85	<0.001	0.72	0.57–0.90	0.005	0.95	0.94–0.97	<0.001	1.11	1.09–1.13	<0.001
2	0.91	0.900.93	<0.001	0.85	0.83–0.86	<0.001	0.78	0.52–1.17	0.23	0.81	0.49–1.33	0.40	0.72	0.70–0.74	<0.001	0.94	0.92–0.97	<0.001
≥ 3	0.86	0.84–0.88	<0.001	0.80	0.77–0.82	<0.001	0.49	0.29–0.83	0.008	0.46	0.26–0.82	0.008	0.55	0.53–0.57	<0.001	0.79	0.76–0.82	<0.001
*AJCC clinical stage*																		
0 (ref for surgery, XRT)																		
1 (ref for SLNB)	1.12	1.11–1.13	<0.001	0.90	0.86–0.94	<0.001	0.66	0.54–0.80	<0.001	0.52	0.18–1.49	0.22						
2	0.43	0.43–0.43	<0.001	0.69	0.66–0.72	<0.001	0.27	0.22–0.33	<0.001	0.33	0.11–0.99	0.05	0.70	0.69–0.70	<0.001	0.64	0.62–0.66	<0.001
*Tumor subtype*																		
HR+ HER2− (ref)																		
HER2+	0.62	0.61–0.63	<0.001	0.88	0.87–0.89	<0.001	0.30	0.25–0.37	<0.001	0.40	0.32–0.50	<0.001	1.00	0.98–1.02	0.98	1.03	1.01–1.05	0.003
HR− HER2−	0.71	0.71–0.72	<0.001	1.01	1.00–1.03	0.04	0.31	0.26–0.38	<0.001	0.53	0.41–0.68	<0.001	0.94	0.93–0.96	<0.001	1.07	1.05–1.09	<0.001
*Tumor grade*																		
Well-differentiated (ref)																		
Moderately differentiated	0.68	0.67–0.68	<0.001	0.75	0.75–0.76	<0.001	0.66	0.54–0.80	<0.001	0.73	0.56–0.97	0.03	0.97	0.96–0.99	<0.001	1.00	0.99–1.01	0.96
Poorly differentiated	0.52	0.52–0.53	<0.001	0.73	0.72–0.74	<0.001	0.37	0.30–0.45	<0.001	0.57	0.42–0.76	<0.001	0.91	0.90–0.93	<0.001	0.91	0.89–0.92	<0.001
*Clinical tumor stage*																		
0 (ref)																		
1	1.12	1.12–1.13	<0.001	1.67	1.61–1.74	<0.001	0.62	0.51–0.75	<0.001	1.19	0.46–3.08	0.72						
2	0.44	0.44–0.44	<0.001	0.97	0.93–1.01	0.14	0.30	0.25–0.37	<0.001	1.24	0.46–3.36	0.68	0.77	0.76–0.78	<0.001	1.14	1.11–1.17	<0.001
3													0.48	0.46–0.49	<0.001	0.74	0.71–0.78	<0.001
*Clinical nodal stage*																		
0 (ref)																		
1	0.37	0.37–0.37	<0.001	0.63	0.62–0.64	<0.001	0.39	0.33–0.47	<0.001	0.92	0.68–1.23	0.55						
*Low SES Institution*																		
No (ref)																		
Yes	0.78	0.78–0.79	<0.001	0.83	0.82–0.84	<0.001	0.49	0.41–0.58	<0.001	0.85	0.66–1.09	0.21	0.76	0.75–0.77	<0.001	0.85	0.84–0.87	<0.001
*Low SES patient*																		
No (ref)																		
Yes	0.84	0.84–0.85	<0.001	0.94	0.93–0.95	<0.001	0.42	0.37–0.48	<0.001	0.47	0.40–0.57	<0.001	0.82	0.81–0.83	<0.001	0.83	0.81–0.84	<0.001

*OR* odds ratio; *CI* confidence interval; *ref* reference variable; *SES* socioeconomic status; *AJCC* American Joint Committee on Cancer

### Sentinel Lymphadenectomy

Patients with AJCC clinical Stage I or Stage II disease were identified from the main cohort, which represents patients eligible for sentinel lymphadenectomy (SLNB) in the nonneoadjuvant setting based on the 2018 NAPBC criteria. This cohort was composed of 1.07 million patients, 83,261 (8%) of whom were treated at low SES institutions (Supplementary Table 2). Overall, the unadjusted rate of SLNB was 81%, with 14% of patients going on to have a completion axillary lymph node dissection (ALND). Similar to BCS, the highest rate of SLNB was among nonlow SES patients being treated at non-low SES institutions and the lowest was among low-SES patients being treated at low SES institutions (82.1% vs. 75.5%, *p* < 0.001; Fig. 1).

Both low institution and patient SES were associated with lower odds of SLNB on univariate (OR 0.76, *p* < 0.001; OR 0.82, *p* < 0.001, respectively) and multivariable analysis when adjusted for clinicopathologic features (OR 0.85, *p* < 0.001; OR 0.83, *p* < 0.001, respectively; Table 3).

### Adjuvant Radiotherapy

Patients < 70 years old at the time of diagnosis were selected from the BCS cohort as a group of patients for whom adjuvant radiation (XRT) is standard. This cohort was composed of 1.7 million patients, 133,137 (8%) of whom were treated at low SES institutions (Supplementary Table 1). Significant differences in patient demographics and tumor characteristics persisted when comparing groups between and within institution type (all *p* < 0.001). At least 80% of patients in each subgroup underwent radiation within 365 days with unadjusted rates being 3% lower in low SES patients regardless of institution type (Fig. 1). When comparing nonlow SES patients, unadjusted rated of XRT within 365 days were nearly equal at low SES institutions (83.5% vs. 82.5%).

Both low institution and patient SES were associated with lower odds of undergoing XRT within 365 days on univariate analysis (OR 0.49, *p* < 0.001; OR 0.42, *p* < 0.001, respectively; Table 3). However, on multivariable analysis, when adjusted for clinicopathologic features, institution SES was no longer independently associated with the odds of undergoing adjuvant radiation (OR 0.85, *p* = 0.21), whereas low patient SES remained independently associated with lower odds of undergoing adjuvant radiation (OR 0.47, *p* < 0.001).

### Adjuvant Endocrine Therapy

Patients with AJCC T1c, Stage II, or Stage III hormone-receptor positive breast cancer were identified from the main data file. This cohort, eligible for adjuvant endocrine therapy (ETX), was composed of 680,843 patients, 56,018 (8%) of whom were treated at low SES institution (Supplementary Table 2). Overall, 81% of patients received ETX and 75% started therapy within 365 days. Unadjusted rates of receipt of ETX were similar when stratified by SES institution (low: 80% vs. nonlow: 81%), with equivalent rates of initiating therapy within 365 days (both 75%). The largest difference in unadjusted rates of receipt of ETX were noted between patient SES groups at low SES institutions (Fig. 1). Specifically, 78% of nonlow SES patients received ETX within 365 days, whereas the rate among low SES patients was 74% (*p* < 0.001).

Both low institution and patient SES were associated with lower odds of receipt of ETX within 365 days on univariate (OR 0.62, *p* < 0.001; OR 0.65, *p* < 0.001, respectively) and multivariable analysis when adjusted for clinicopathologic features (HR 0.79, *p* < 0.001; HR 0.79, *p* < 0.001, respectively; Table 4).

**Table 4:** Univariate and multivariable analyses of the odds of patients undergoing guideline concordant care according to adjuvant hormone therapy and chemotherapy standards

	Adjuvant hormone therapy						Adjuvant chemotherapy					
	Univariate			Multivariable			Univariate			Multivariable		
	OR	95% CI	*p*	OR	95% CI	*p*	OR	95% CI	*p*	OR	95% CI	*p*
*Age*	1.03	1.03–1.03	<0.001	1.02	1.02–1.03	<0.001	0.99	0.98–0.99	<0.001	0.99	0.98–1.00	0.002
*Race*												
White (ref)												
Black	0.52	0.48–0.55	<0.001	0.60	0.53–0.68	<0.001	0.56	0.50–0.63	<0.001	0.65	0.56–0.75	<0.001
Other	0.90	0.80–1.01	0.07	0.91	0.75–1.11	0.35	0.88	0.67–1.16	0.35	0.9	0.66–1.24	0.53
*Institution type*												
Community Cancer Center (ref)												
Comprehensive community cancer program	1.07	0.97–1.19	0.18	1.13	0.94–1.36	0.19	1.31	1.05–1.63	0.02	1.33	1.05–1.68	0.02
Academic/research program	0.88	0.80–0.98	0.02	1.09	0.91–1.32	0.36	1.05	0.84–1.31	0.7	1.18	0.93–1.50	0.18
Integrated network cancer program	1.09	0.98–1.22	0.13	1.26	1.03–1.54	0.03	1.25	0.99–1.59	0.07	1.22	0.94–1.57	0.14
*Charlson/Deyo score*												
0 (ref)												
1	1.07	1.00–1.16	0.07	0.91	0.79–1.05	0.19	0.83	0.70–0.98	0.03	0.93	0.77–1.13	0.47
2	1.10	0.95–1.29	0.21	0.81	0.63–1.04	0.1	0.53	0.39–0.73	<0.001	0.7	0.49–1.01	0.06
≥3	1.57	1.21–2.04	<0.001	1.00	0.70–1.42	1.00	0.46	0.29–0.72	<0.001	0.88	0.49–1.58	0.67
*AJCC pathologic stage*												
1 (ref)												
2	0.44	0.42–0.47	<0.001	0.78	0.62–0.99	0.04	0.93	0.81–1.06	0.25			
3	0.30	0.28–0.32	<0.001	0.98	0.70–1.38	0.91	1.11	0.93–1.31	0.25			
*HER2 subtype*												
HER2− (ref)												
HER2+	0.26	0.24–0.29	<0.001	0.29	0.26–0.33	<0.001	1.03	0.79–1.34	0.82			
*Tumor grade*												
Well-differentiated (ref)												
Moderately differentiated	0.70	0.64–0.75	<0.001	0.85	0.73–0.99	0.04	1.23	0.76–2.01	0.40			
Poorly differentiated	0.36	0.34–0.39	<0.001	0.58	0.49–0.68	<0.001	1.29	0.81–2.06	0.29			
*Tumor stage*												
0 (ref)												
1a/b	0.37	0.20–0.70	0.002	0.15	0.02–1.11	0.06	0.17	0.04–0.70	0.01	0.24	0.06–0.98	0.05
1c	0.82	0.44–1.53	0.54	0.09	0.01–0.67	0.02	0.13	0.03–0.52	0.004	0.24	0.06–0.96	0.04
2	0.42	0.23–0.79	0.007	0.08	0.01–0.56	0.01	0.11	0.03–0.45	0.002	0.20	0.05–0.80	0.02
3	0.38	0.20–0.71	0.002	0.07	0.01–0.51	0.01	0.11	0.03–0.45	0.002	0.19	0.05–0.79	0.02
4	0.42	0.22–0.81	0.009	0.10	0.01–0.72	0.02	0.22	0.05–0.92	0.04	0.38	0.09–1.64	0.19
*Nodal stage*												
0 (ref)												
1	0.53	0.50–0.57	<0.001	0.65	0.57–0.74	<0.001	1.04	0.88–1.22	0.66	0.96	0.80–1.15	0.68
2	0.34	0.32–0.37	<0.001	0.37	0.28–0.48	<0.001	1.26	1.01–1.56	0.04	1.20	0.95–1.51	0.13
3	0.31	0.28–0.34	<0.001	0.29	0.21–0.39	<0.001	1.90	1.34–2.70	<0.001	1.83	1.26–2.67	0.002
*Low SES institution*												
No (ref)												
Yes	0.62	0.58–0.67	<0.001	0.79	0.68–0.92	0.002	0.59	0.51–0.68	<0.001	0.73	0.61–0.88	<0.001
*Low SES patient*												
No (ref)												
Yes	0.65	0.62–0.69	<0.001	0.79	0.71–0.88	<0.001	0.56	0.51–0.63	<0.001	0.68	0.59–0.77	<0.001

*OR* odds ratio; *CI* confidence interval; *ref* reference variable; *SES* socioeconomic status; *AJCC* American Joint Committee on Cancer

### Adjuvant Chemotherapy

Patients under the age of 70 with AJCC T1c, Stage II, or Stage III hormone receptor-negative breast cancer were identified from the main data file, who represent patients for whom adjuvant chemotherapy (CTX) should be considered according to NAPBC standards. A total of 110,899 were identified, 12,091 (11%) of whom were treated at low SES institutions (Supplementary Table 3). Overall, 89% of patients underwent CTX, and 82% received CTX within 120 days. A similar proportion of patients received CTX when stratified by institution SES overall (low: 88% vs. nonlow 89%) and within 120 days (low: 83% vs. nonlow: 82%). Similar to ETX, the largest difference in unadjusted rates of receipt of chemotherapy were noted between patient SES groups within 120 days (Fig. 1). Nonlow SES patients treated at low SES institutions had the highest rate (85%), whereas low SES patients treated at nonlow SES institutions had the lowest rate (80%).

Both low institution and patient SES were associated with lower odds of receipt of CTX within 120 days on univariate (OR 0.59, *p* < 0.001; OR 0.56, *p* < 0.001, respectively) and multivariable analysis when adjusted for clinicopathologic features (OR 0.73, *p* < 0.001; OR 0.68, *p* < 0.001, respectively; Table 4).

## Discussion

Our previous findings show that an institution’s socioeconomic status has an impact on the achievement of multidisciplinary standards for quality breast cancer care.^8^ The current study adds to these findings and demonstrates that SES status at both the patient and institution level are independently associated with lower odds of achieving specific standard-compliant treatment for all NAPBC standards, except for XRT. Moreover, it denotes that surgery may be more readily accessible or more commonly offered to patients of low SES at low SES institutions compared with the receipt of adjuvant treatments.

Our findings have several implications for understanding the complicated interplay of factors associated with quality breast cancer care. For instance, whereas nonlow SES patients typically have better quality outcomes and healthcare perceptions, being treated at low SES institutions may result in poorer patient outcomes. This could be because most low-SES institutions are public hospitals that serve disadvantaged communities and experience strains on hospital systems and resources.^11^ It also may be due to reduced efficiency within lower-SES institutions, especially in how it relates to resource-intensive, yet essential, multidisciplinary care for patients with breast cancer.^12,13^ However, given their likelihood of higher education and insurance status, based on this study’s demographic data, nonlow SES patients may be able to more effectively navigate these barriers and continue to have improved overall outcomes. These institutions may have a motivation to provide additional resources, or in other ways treat nonlow SES patients differently, given that their insurance status creates powerful incentives for hospitals to focus their services on them.^14^

Conversely, patients of low-SES that receive treatment from nonlow SES institutions may find themselves in facilities with readily available resources, a diverse set of in-house subspecialists, and multidisciplinary care teams that ensure patient-centered care. Previous studies have shown that low-SES institutions (such as safety-net hospitals in large, metropolitan cities) have low hospital quality owing to lack of financial and human resources and may be located in economically disadvantaged neighborhoods.^15^ Nevertheless, despite treatment at nonlow SES institutions, low-SES patients may continue to face social (e.g., transportation issues, difficulty scheduling time from work) or economic (e.g., medication copayments) challenges that make it difficult to achieve long term care goals. One phenomenon, known as financial toxicity (FT), can affect patients of both statuses. Described as the negative impact caused by the financial burden of extensive medical care (and how it affects patients’ physical/mental health and their economic security), FT affects low-SES patients at disproportionate rates.^16,17^ This is especially concerning for low-SES patients treated at low-SES institutions, which may not have the resources to offset the financial burden that comes with comprehensive cancer care, leaving patients to forego critical portions of their care. This has the potential for compounding disparities and poorer outcomes in this patient population.

While not explicitly linked to our definition of low SES, an additional factor that demonstrated significant disadvantage throughout all five measures in our analysis was race. Black patients experience higher mortality from breast cancer, despite a lower incidence rate compared to White patients.^18^ While the reasons for this are complex and multifactorial, one explanation is that Black women tend to be diagnosed at more advanced stages and with more aggressive breast cancer subtypes and tumor grades.^19^ Our multivariable analyses adjust for these factors and still show that race is an independent factor associated with lower odds of guideline-concordant care. The more advanced disease may, however, account for the higher unadjusted rate we found for radiotherapy in breast conservation patients. In terms of quality of care, Black Americans are more likely to receive care at facilities with lower quality metric scores or less than average funding and have negative perceptions of the care they receive.^19,20^ Black women also have significantly lower rates of breast cancer screening (a datapoint historically not collected by the NCDB) leading to greater chance of late-stage diagnosis.^21–23^ In this analysis, a larger proportion of Black patients were treated at low-SES institutions for all five treatment standards, regardless of the patient’s SES. However, it is still important to consider patient SES, given its high correlation with race and ethnicity.^18,24^ For example, a study by Nayyar et al.^25^ showed that owing to significant disparities in care, Black patients from communities that had higher poverty levels, lack of education, and no insurance were more likely to receive unwarranted ALND rather than SLNB in situations where the latter would have been indicated. Several studies have also demonstrated that Black patients experienced relevant delays in initiating CTX and XRT compared with White patients owing to the socioeconomic disadvantages discussed previously.^26,27^ Given the complex interaction between race and SES, further understanding of the root causes of and targeted interventions for dismantling these disparities are needed.

Tumor subtype and grade also impacted the use of the treatment measures we studied in this analysis. These factors also differed between institution type, making our multivariable analysis critical to understanding this complex association. We found that patients treated at low-SES institutions more commonly presented with poorly differentiated cancer grades and triple-negative cancer, regardless of patient SES. This may be owing to inherent tumor characteristics (such as subtype and grade) that copresent with factors associated with SES. Increasing knowledge of epigenetics and tumor genomics seen between different racial and ethnic groups may clarify this phenomenon. For instance, Black women are more likely to present with genetic differences that may explain the prevalence of hormone-negative or triple-negative cancer subtypes, especially compared with White women.^24,28–30^ Additional understanding of these factors will be an important piece of this complicated puzzle of sociodemographic features, breast cancer risk, and outcomes.

Finally, we found that guideline-concordant use of XRT was the only standard that demonstrated no significant differences between institution and patient SES. Parekh et al.^31^ found that race, ethnicity, insurance status, education level, and age were factors associated with the receipt of XRT in a recent analysis of the NCDB, which suggests that SES is a factor associated with the use of XRT. One potential explanation for the difference in these findings is that the NAPBC XRT standard examines only patients who were candidates for and underwent BCS. In our cohort, we found disparities in performance of BCS among low SES institution and patient SES; thus the XRT analysis cohort is already enriched for non-low SES patients and institutions making differences in XRT less prevalent. More granular analysis of the XRT regimen chosen and completion of all recommended fractions is an area for further analysis to understand whether these groups are truly equivalent.

The current study adds to the literature defining disparities for low SES breast cancer patients and prompts care teams, accreditation organizations, and lawmakers to conduct and fund studies focused on root causes and interventions that can help reduce these disparities. Once such intervention is increasing patient navigation resources in low SES institutions, which may often be absent in smaller, less resourced settings. A recent meta-analysis of navigation in cancer care found increases in treatment initiation, adherence, and improvements in quality care indicators when navigation is present.^32^ Navigators should not only be trained in clinical pathways but also be culturally competent and responsive to the setting in which they work and be connected to local social services that address the specific challenges of their patients.

### Strengths and Limitations

National database studies have several inherent strengths and limitations but are critical to understand the landscape of breast cancer care provided in this country. Our use of the NCDB, which is a large, diverse dataset that includes detailed clinical and demographic information, permitted this analysis and represents a major strength of this study. The large number of patients with data pertinent to NAPBC standards allowed us to construct robust multivariable models to assess the impact of both an individual’s SES status, as well as that of the institution in which they were treated. A limitation of the study is that while many datapoints are available, we lack some granular data that may help explain the trends and other factors that may contribute to achieving SCT. It is also important to note that basing an institution’s SES on the percentage of Medicaid patients is a proxy measurement, allowing us to categorize the data in a meaningful manner. However, given the deidentified nature of the institutions, there is no way to determine if a low SES institution is actually a hospital with low resource availability (or an actual safety-net hospital).

Furthermore, all institutions contributing data to the NCDB are accredited as CoC institutions, and these groups may already be more likely to adhere to evidence-based practices. Therefore, the results of this analysis may underestimate the true degree of disparity experienced by low SES patients treated in nonaccredited institutions. Case volume is a well-known factor contributing to quality of care, and while we eliminated centers with fewer than 100 cases, some low-volume centers may still be included and may skew our results. This is not a factor that we corrected for because the number of surgeons at each center is unknown. However, future analyses, including these low-volume centers, may be helpful to determine the interaction of SES factors with case volume. Additionally, while we did not explicitly account for the location of these low-SES institutions, previous work has focused on neighborhood-level SES and breast cancer outcomes.^15,21,33^ Understanding the demographics at the neighborhood level, and correlating to site of care, distance traveled, and cancer outcomes are an intriguing area for future study. Finally, the NCDB does not include data related to disease-specific outcomes and the impact of NAPBC accreditation on recurrence, and survival is still under investigation. This also remains an area for future work and would help to put the treatment differences we report here in the context of patient outcomes.

## Conclusions

This study found that both patient and institution SES are independent predictors of breast cancer quality outcomes for all NAPBC standards, except for adjuvant radiation therapy. Notably, low SES patients had lower rates of standard-compliant treatment regardless of the institution’s SES. The social and financial resources of institutions and patients themselves likely provide a synergistic effect that impacts almost all aspects of multidisciplinary breast cancer care. Further research and targeted interventions are needed to improve standard-compliant treatment and prevent disparities in breast cancer care.

## Supplementary Information

Below is the link to the electronic supplementary material.

Supplementary file1 (DOCX 63 KB)
