# Supplementary material for: Low Socioeconomic Status at the Patient and Institutional Level Impact Achievement of Breast Cancer Quality Standards
Source: Ann Surg Oncol. 2025 Jun 2;32(12):8644–55. doi: 10.1245/s10434-025-17529-w (PMC12534247; doi:10.1245/s10434-025-17529-w)
Supplement: Supplementary file 1 — Supplementary file1 (DOCX 63 KB) [file 10434_2025_17529_MOESM1_ESM.docx]

**Supplementary Table 1**: Demographic and clinicopathologic features of Clinical Stage 0, I, and II patients (BCS Analysis Cohort), and the subset of these patients <70 years old (Adjuvant Radiation Analysis Cohort) stratified by institution and patient socioeconomic status.

|  |  | |  | **BCS Analysis Cohort** | | | | **Adjuvant Radiation Analysis Cohort** | | | |
| --- | --- | --- | --- | --- | --- | --- | --- | --- | --- | --- | --- |
|  |  | |  | **Non-low SES Institutions** | | **Low SES Institutions** | | **Non-low SES Institutions** | | **Low SES Institutions** | |
|  |  | |  | ***Non-low SES patients*** | ***Low SES patients*** | ***Non-low SES patients*** | ***Low SES patients*** | ***Non-low SES patients*** | ***Low SES patients*** | ***Non-low SES patients*** | ***Low SES patients*** |
| **n** |  | |  | 1,696,844 | 474,181 | 78,342 | 101,774 | 1,201,632 | 356,327 | 55,450 | 77,687 |
| **Age** | | |  | 61.8 ± 12.9 | 60.2 ± 13.0 | 62.1 ±12.6 | 60.1 ± 12.7 | 55.5 ± 9.2 | 55.6 ± 9.5 | 56.0 ± 9.3 | 54.9 ± 9.4 |
| **Race** | | | |  |  |  |  |  |  |  |  |
|  | White | | | 1,484,841 (87.5) | 342,121 (72.1) | 63,368 (80.9) | 66,324 (65.2) | 1,033,936 (86.0) | 252,294 (70.8) | 43,811 (79.0) | 49,260 (63.4) |
|  | Black | | | 116,700 (6.9) | 95,264 (20.1) | 12,257 (15.6) | 30,689 (30.2) | 90,438 (7.5) | 73,418 (20.6) | 9,382 (16.9) | 24,364 (31.4) |
|  | Other | | | 80,097 (4.7) | 31,856 (6.7) | 2,277 (2.9) | 3,885 (3.8) | 65,450 (5.4) | 26,661 (7.5) | 1,923 (3.5) | 3,377 (4.3) |
|  | Unknown | | | 15,206 (0.9) | 4,940 (1.0) | 440 (0.6) | 876 (0.9) | 11,808 (1.0) | 3,954 (1.1) | 334 (0.6) | 686 (0.9) |
| **Insurance status** | | | |  |  |  |  |  |  |  |  |
|  | Not insured | | | 0 (0.0) | 23,806 (5.0) | 0 (0.0) | 11,790 (11.6) | 0 (0.0) | 22,133 (6.2) | 0 (0.0) | 11,219 (14.4) |
|  | Private insurance | | | 976,984 (57.6) | 176,388 (37.2) | 40,991 (52.3) | 32,162 (31.6) | 926,872 (77.1) | 164,258 (46.1) | 38,604 (69.6) | 30,059 (38.7) |
|  | Medicaid | | | 0 (0.0) | 106,742 (22.5) | 0 (0.0) | 19,294 (19.0) | 0 (0.0) | 99,630 (28.0) | 0 (0.0) | 18,331 (23.6) |
|  | Medicare | | | 680,895 (40.1) | 157,402 (33.2) | 33,996 (43.4) | 35,551 (34.9) | 242,823 (20.2) | 62,246 (17.5) | 13,986 (25.2) | 15,531 (20.0) |
|  | Other Government | | | 17,522 (1.0) | 3,538 (0.7) | 1,996 (2.5) | 973 (1.0) | 15,440 (1.3) | 3,111 (0.9) | 1,811 (3.3) | 865 (1.1) |
|  | Unknown | | | 21,443 (1.3) | 6,305 (1.3) | 1,359 (1.7) | 2,004 (2.0) | 16,497 (1.4) | 4,949 (1.4) | 1,049 (1.9) | 1,682 (2.2) |
| **Percent No High School Degree** | | | |  |  |  |  |  |  |  |  |
|  | 17.6% or more | | | 0 (0.0) | 281,049 (59.3) | 0 (0.0) | 71,864 (70.6) | 0 (0.0) | 203,748 (57.2) | 0 (0.0) | 53,288 (68.6) |
|  | 10.9% - 17.5% | | | 331,581 (19.5) | 108,414 (22.9) | 27,251 (34.8) | 20,277 (19.9) | 231,405 (19.3) | 79,929 (22.4) | 18,967 (34.2) | 15,756 (20.3) |
|  | 6.3% - 10.8% | | | 524,496 (30.9) | 45,339 (9.6) | 22,525 (28.8) | 5,148 (5.1) | 366,974 (30.5) | 37,275 (10.5) | 16,081 (29.0) | 4,438 (5.7) |
|  | Less than 6.3% | | | 592,307 (34.9) | 23,159 (4.9) | 16,542 (21.1) | 1,721 (1.7) | 426,720 (35.5) | 20,261 (5.7) | 12,048 (21.7) | 1,563 (2.0) |
|  | Unknown | | | 248,460 (14.6) | 16,220 (3.4) | 12,024 (15.3) | 2,764 (2.7) | 176,533 (14.7) | 15,114 (4.2) | 8,354 (15.1) | 2,642 (3.4) |
| **Median Income Quartiles** | | | |  |  |  |  |  |  |  |  |
|  | Less than $40,227 | | | 0 (0.0) | 232,583 (49.0) | 0 (0.0) | 69,287 (68.1) | 0 (0.0) | 162,144 (45.5) | 0 (0.0) | 50,682 (65.2) |
|  | $40,227 - $50,353 | | | 258,641 (15.2) | 99,138 (20.9) | 24,288 (31.0) | 18,590 (18.3) | 171,862 (14.3) | 75,814 (21.3) | 16,541 (29.8) | 14,734 (19.0) |
|  | $50,354 - $63,332 | | | 370,900 (21.9) | 75,288 (15.9) | 22,623 (28.9) | 7,459 (7.3) | 254,200 (21.2) | 59,848 (16.8) | 16,063 (29.0) | 6,333 (8.2) |
|  | $63,333 or more | | | 817,016 (48.1) | 50,109 (10.6) | 19,222 (24.5) | 3,410 (3.4) | 597,854 (49.8) | 42,792 (12.0) | 14,365 (25.9) | 3,103 (4.0) |
|  | Unknown | | | 250,287 (14.8) | 17,063 (3.6) | 12,209 (15.6) | 3,028 (3.0) | 177,716 (14.8) | 15,729 (4.4) | 8,481 (15.3) | 2,835 (3.6) |
| **Institution Type** | | | |  |  |  |  |  |  |  |  |
|  | Community Cancer Center | | | 108,331 (6.4) | 34,076 (7.2) | 4,585 (5.9) | 12,194 (12.0) | 69,217 (5.8) | 24,299 (6.8) | 2,949 (5.3) | 8,525 (11.0) |
|  | Comprehensive Community Cancer Program | | | 695,904 (41.0) | 197,924 (41.7) | 25,401 (32.4) | 29,605 (29.1) | 474,980 (39.5) | 142,142 (39.9) | 16,576 (29.9) | 20,702 (26.6) |
|  | Academic/Research Program | | | 447,430 (26.4) | 128,856 (27.2) | 37,510 (47.9) | 47,557 (46.7) | 329,608 (27.4) | 99,966 (28.1) | 27,644 (49.9) | 38,243 (49.2) |
|  | Integrated Network Cancer Program | | | 383,965 (22.6) | 89,231 (18.8) | 7,913 (10.1) | 7,354 (7.2) | 266,613 (22.2) | 65,826 (18.5) | 5,348 (9.6) | 5,153 (6.6) |
|  | Unknown | | | 61,214 (3.6) | 24,094 (5.1) | 2,933 (3.7) | 5,064 (5.0) | 61,214 (5.1) | 24,094 (6.8) | 2,933 (5.3) | 5,064 (6.5) |
| **Charlson/Deyo Score** | | | |  |  |  |  |  |  |  |  |
|  | 0 | |  | 1,437,780 (84.7) | 384,689 (81.1) | 64,573 (82.4) | 81,237 (79.8) | 1,054,512 (87.8) | 297,707 (83.5) | 47,343 (85.4) | 63,761 (82.1) |
|  | 1 | |  | 198,990 (11.7) | 67,185 (14.2) | 10,477 (13.4) | 15,506 (15.2) | 119,247 (9.9) | 45,324 (12.7) | 6,400 (11.5) | 10,812 (13.9) |
|  | 2 | |  | 41,878 (2.5) | 15,375 (3.2) | 2,248 (2.9) | 3,356 (3.3) | 20,458 (1.7) | 9,370 (2.6) | 1,199 (2.2) | 2,117 (2.7) |
|  | ≥3 | | | 18,196 (1.1) | 6,932 (1.5) | 1,044 (1.3) | 1,675 (1.6) | 7,415 (0.6) | 3,926 (1.1) | 508 (0.9) | 997 (1.3) |
| **AJCC Clinical Stage** | | | |  |  |  |  |  |  |  |  |
|  | 0 |  | | 413,551 (24.4) | 109,194 (23.0) | 17,279 (22.1) | 21,675 (21.3) | 318,620 (26.5) | 85,823 (24.1) | 13,000 (23.4) | 17,130 (22.1) |
|  | 1 |  | | 923,136 (54.4) | 237,359 (50.1) | 42,213 (53.9) | 49,516 (48.7) | 620,621 (51.6) | 169,724 (47.6) | 28,593 (51.6) | 35,964 (46.3) |
|  | 2 |  | | 360,157 (21.2) | 127,628 (26.9) | 18,850 (24.1) | 30,583 (30.0) | 262,391 (21.8) | 100,780 (28.3) | 13,857 (25.0) | 24,593 (31.7) |
| **Tumor subtype** | | | |  |  |  |  |  |  |  |  |
|  | HR+ HER2- | | | 791,616 (46.7) | 209,730 (44.2) | 36,941 (47.2) | 44,263 (43.5) | 529,152 (44.0) | 151,565 (42.5) | 24,901 (44.9) | 32,463 (41.8) |
|  | HER2+ | | | 133,763 (7.9) | 42,431 (8.9) | 7,168 (9.1) | 10,215 (10.0) | 103,523 (8.6) | 34,900 (9.8) | 5,473 (9.9) | 8,367 (10.8) |
|  | HR- HER2- | | | 101,924 (6.0) | 36,031 (7.6) | 5,934 (7.6) | 9,126 (9.0) | 75,318 (6.3) | 28,884 (8.1) | 4,475 (8.1) | 7,466 (9.6) |
|  | Unknown | | | 669,541 (39.5) | 185,989 (39.2) | 28,299 (36.1) | 38,170 (37.5) | 493,639 (41.1) | 140,978 (39.6) | 20,601 (37.2) | 29,391 (37.8) |
| **Tumor Grade** | | | |  |  |  |  |  |  |  |  |
|  | Well-differentiated | | | 362,336 (21.4) | 90,001 (19.0) | 16,149 (20.6) | 18,560 (18.2) | 240,259 (20.0) | 62,863 (17.6) | 10,629 (19.2) | 13,221 (17.0) |
|  | Moderately-differentiated | | | 689,287 (40.6) | 186,052 (39.2) | 30,644 (39.1) | 38,441 (37.8) | 473,509 (39.4) | 135,461 (38.0) | 21,092 (38.0) | 28,393 (36.5) |
|  | Poorly differentiated | | | 453,636 (26.7) | 141,745 (29.9) | 21,847 (27.9) | 31,687 (31.1) | 343,874 (28.6) | 114,052 (32.0) | 16,602 (29.9) | 25,852 (33.3) |
|  | Unknown | | | 191,585 (11.3) | 56,383 (11.9) | 9,702 (12.4) | 13,086 (12.9) | 143,990 (12.0) | 43,951 (12.3) | 7,127 (12.9) | 10,221 (13.2) |
| **Clinical tumor stage** | | | |  |  |  |  |  |  |  |  |
|  | 0 | |  | 424,703 (25.0) | 112,083 (23.6) | 17,768 (22.7) | 22,268 (21.9) | 327,316 (27.2) | 88,131 (24.7) | 13,380 (24.1) | 17,597 (22.7) |
|  | 1 | |  | 931,199 (54.9) | 242,085 (51.1) | 42,725 (54.5) | 51,051 (50.2) | 630,127 (52.4) | 174,262 (48.9) | 29,126 (52.5) | 37,391 (48.1) |
|  | 2 | |  | 340,942 (20.1) | 120,013 (25.3) | 17,849 (22.8) | 28,455 (28.0) | 244,189 (20.3) | 93,934 (26.4) | 12,944 (23.3) | 22,699 (29.2) |
| **Clinical nodal stage** | | | |  |  |  |  |  |  |  |  |
|  | 0 | |  | 1571140 (92.6) | 425,869 (89.8) | 71,374 (91.1) | 90,055 (88.5) | 1,102,968 (91.8) | 315,961 (88.7) | 49,997 (90.2) | 67,799 (87.3) |
|  | 1 | |  | 125704 (7.4) | 48,312 (10.2) | 6,968 (8.9) | 11,719 (11.5) | 98,664 (8.2) | 40,366 (11.3) | 5,453 (9.8) | 9,888 (12.7) |
| **Surgery** | | | |  |  |  |  |  |  |  |  |
|  | None | | | 59232 (3.5) | 21,966 (4.6) | 3,616 (4.6) | 5,987 (5.9) | 31,395 (2.6) | 13,694 (3.8) | 2,023 (3.6) | 4,012 (5.2) |
|  | BCS | | | 1059905 (62.5) | 278,631 (58.8) | 44,564 (56.9) | 55,848 (54.9) | 725,265 (60.4) | 205,851 (57.8) | 30,888 (55.7) | 42,382 (54.6) |
|  | Mastectomy | | | 572739 (33.8) | 171,823 (36.2) | 29,913 (38.2) | 39,522 (38.8) | 441,364 (36.7) | 135,439 (38.0) | 22,351 (40.3) | 30,969 (39.9) |
|  | Unknown | | | 4968 (0.3) | 1,761 (0.4) | 249 (0.3) | 417 (0.4) | 3,608 (0.3) | 1,343 (0.4) | 188 (0.3) | 324 (0.4) |
| ***Patients undergoing BCS only:*** | | | |  |  |  |  |  |  |  |  |
|  | **Radiation** | | |  |  |  |  |  |  |  |  |
|  |  | | No |  |  |  |  | 98,905 (13.6) | 30,749 (14.9) | 4,180 (13.5) | 6,252 (14.8) |
|  |  | | Yes |  |  |  |  | 609,370 (84.0) | 169,256 (82.2) | 26,083 (84.4) | 34,980 (82.5) |
|  |  | | Unknown |  |  |  |  | 16,990 (2.3) | 5,846 (2.8) | 625 (2.0) | 1,150 (2.7) |
|  | **Radiation within 365 days** | | |  |  |  |  |  |  |  |  |
|  |  | | No |  |  |  |  | 549 (0.1) | 339 (0.2) | 37 (0.1) | 106 (0.3) |
|  |  | | Yes |  |  |  |  | 598,572 (82.5) | 165,143 (80.2) | 25,783 (83.5) | 34,265 (80.8) |
|  |  | | Unknown |  |  |  |  | 126,144 (17.4) | 40,369 (19.6) | 5,068 (16.4) | 8,011 (18.9) |

Note: All comparisons between and within institution SES categories for each analysis cohort are statistically significant with a p<0.001.SES: socioeconomic status; AJCC: American Joint Committee on Cancer; HR: hormone receptor

**Supplementary Table 2:** Demographic and clinicopathologic features of Clinical Stage I and II patients (Sentinel Lymphadenectomy Analysis Cohort) stratified by institution and patient socioeconomic status.

|  |  | **Non-low SES Institutions** | | **Low SES Institutions** | |
| --- | --- | --- | --- | --- | --- |
|  |  | ***Non-low SES patients*** | ***Low SES patients*** | ***Non-low SES patients*** | ***Low SES patients*** |
| **n** |  | 776,522 | 210,666 | 37,569 | 45,692 |
| **Age** | | 63.2 ± 12.8 | 61.1 ± 13.0 | 63.3 ± 12.4 | 61.1 ± 12.7 |
| **Race** | |  |  |  |  |
|  | White | 683,095 (88.0) | 154,790 (73.5) | 30,831 (82.1) | 30,964 (67.8) |
|  | Black | 50,699 (6.5) | 39,076 (18.5) | 5,561 (14.8) | 12,595 (27.6) |
|  | Other | 36,864 (4.7) | 14,832 (7.0) | 996 (2.7) | 1,763 (3.9) |
|  | Unknown | 5,864 (0.8) | 1,968 (0.9) | 181 (0.5) | 370 (0.8) |
| **Insurance status** | |  |  |  |  |
|  | Not insured | 0 (0.0) | 9,545 (4.5) | 0 (0.0) | 4,945 (10.8) |
|  | Private insurance | 410,117 (52.8) | 71,311 (33.9) | 18,163 (48.3) | 13,563 (29.7) |
|  | Medicaid | 0 (0.0) | 50,484 (24.0) | 0 (0.0) | 8,527 (18.7) |
|  | Medicare | 349,012 (44.9) | 75,145 (35.7) | 18,080 (48.1) | 17,677 (38.7) |
|  | Other Government | 8,453 (1.1) | 1,595 (0.8) | 915 (2.4) | 447 (1.0) |
|  | Unknown | 8,940 (1.2) | 2,586 (1.2) | 411 (1.1) | 533 (1.2) |
| **Percent No High School Degree** | |  |  |  |  |
|  | 17.6% or more | 0 (0.0) | 123,031 (58.4) | 0 (0.0) | 32,077 (70.2) |
|  | 10.9% - 17.5% | 148,453 (19.1) | 48,050 (22.8) | 12,714 (33.8) | 9,128 (20.0) |
|  | 6.3% - 10.8% | 233,077 (30.0) | 20,554 (9.8) | 10,563 (28.1) | 2,295 (5.0) |
|  | Less than 6.3% | 262,469 (33.8) | 10,552 (5.0) | 7,786 (20.7) | 797 (1.7) |
|  | Unknown | 132,523 (17.1) | 8,479 (4.0) | 6,506 (17.3) | 1,395 (3.1) |
| **Median Income Quartiles** | |  |  |  |  |
|  | Less than $40,227 | 0 (0.0) | 102,138 (48.5) | 0 (0.0) | 31,320 (68.5) |
|  | $40,227 - $50,353 | 117,151 (15.1) | 44,068 (20.9) | 11,440 (30.5) | 8,164 (17.9) |
|  | $50,354 - $63,332 | 166,946 (21.5) | 33,104 (15.7) | 10,535 (28.0) | 3,136 (6.9) |
|  | $63,333 or more | 359,090 (46.2) | 22,498 (10.7) | 9,004 (24.0) | 1,554 (3.4) |
|  | Unknown | 133,335 (17.2) | 8,858 (4.2) | 6,590 (17.5) | 1,518 (3.3) |
| **Institution Type** | |  |  |  |  |
|  | Community Cancer Center | 52,252 (6.7) | 15,534 (7.4) | 2,195 (5.8) | 5,454 (11.9) |
|  | Comprehensive Community Cancer Program | 317,142 (40.8) | 87,841 (41.7) | 12,625 (33.6) | 13,865 (30.3) |
|  | Academic/Research Program | 209,152 (26.9) | 57,766 (27.4) | 17,790 (47.4) | 20,834 (45.6) |
|  | Integrated Network Cancer Program | 173,188 (22.3) | 39,263 (18.6) | 3,783 (10.1) | 3,439 (7.5) |
|  | Unknown | 24,788 (3.2) | 10,262 (4.9) | 1,176 (3.1) | 2,100 (4.6) |
| **Charlson/Deyo Score** | |  |  |  |  |
|  | 0 | 645,120 (83.1) | 167,977 (79.7) | 30,354 (80.8) | 35,680 (78.1) |
|  | 1 | 97,236 (12.5) | 30,826 (14.6) | 5,323 (14.2) | 7,352 (16.1) |
|  | 2 | 22,538 (2.9) | 7,814 (3.7) | 1,226 (3.3) | 1,709 (3.7) |
|  | ≥3 | 11,628 (1.5) | 4,049 (1.9) | 666 (1.8) | 951 (2.1) |
| **AJCC Clinical Stage** | |  |  |  |  |
|  | 1 | 608,351 (78.3) | 154,000 (73.1) | 28,748 (76.5) | 32,338 (70.8) |
|  | 2 | 168,171 (21.7) | 56,666 (26.9) | 8,821 (23.5) | 13,354 (29.2) |
| **Tumor stage** | |  |  |  |  |
|  | 1 | 583,513 (75.1) | 146,706 (69.6) | 27,415 (73.0) | 30,864 (67.5) |
|  | 2 | 175,195 (22.6) | 57,281 (27.2) | 9,167 (24.4) | 13,067 (28.6) |
|  | 3 | 17,814 (2.3) | 6,679 (3.2) | 987 (2.6) | 1,761 (3.9) |
| **Tumor subtype** | |  |  |  |  |
|  | HR+ HER2- | 595,528 (76.7) | 152,206 (72.2) | 27,350 (72.8) | 31,331 (68.6) |
|  | HER2+ | 82,180 (10.6) | 25,105 (11.9) | 4,389 (11.7) | 5,934 (13.0) |
|  | HR- HER2- | 69,702 (9.0) | 24,160 (11.5) | 4,033 (10.7) | 5,976 (13.1) |
|  | Unknown | 29,112 (3.7) | 9,195 (4.4) | 1,797 (4.8) | 2,451 (5.4) |
| **Tumor Grade** | |  |  |  |  |
|  | Well-differentiated | 199,844 (25.7) | 47,135 (22.4) | 9,384 (25.0) | 9,913 (21.7) |
|  | Moderately-differentiated | 345,692 (44.5) | 90,032 (42.7) | 16,015 (42.6) | 18,697 (40.9) |
|  | Poorly differentiated | 172,314 (22.2) | 55,446 (26.3) | 9,168 (24.4) | 12,922 (28.3) |
|  | Unknown | 58,672 (7.6) | 18,053 (8.6) | 3,002 (8.0) | 4,160 (9.1) |
| **Axillary surgery** | |  |  |  |  |
|  | None | 80,087 (10.3) | 21,796 (10.3) | 3,963 (10.5) | 5,028 (11.0) |
|  | Biopsy | 2,688 (0.3) | 934 (0.4) | 179 (0.5) | 240 (0.5) |
|  | Sentinel lymphadenectomy | 532,038 (68.5) | 136,130 (64.6) | 24,398 (64.9) | 27,725 (60.7) |
|  | SLNB + axillary lymph node dissection | 105,260 (13.6) | 31,203 (14.8) | 5,232 (13.9) | 6,779 (14.8) |
|  | ALND alone | 54,251 (7.0) | 19,774 (9.4) | 3,704 (9.9) | 5,759 (12.6) |
|  | Other/Unknown | 2,198 (0.3) | 829 (0.4) | 93 (0.2) | 161 (0.4) |

Note: All comparisons between and within institution SES categories for each analysis cohort are statistically significant with a p<0.001.SES: socioeconomic status; HR: hormone receptor; SLNB: sentinel lymphadenectomy; ALND: axillary lymph node dissection; AJCC: American Joint Committee on Cancer

**Supplementary Table 3:** Demographic and clinicopathologic features of Pathologic Stage T1c, II and III patients stratified by institution and patient socioeconomic status. Cohorts selected for hormone receptor positivity (Adjuvant Hormone Therapy Analysis Cohort) and <70 with hormone receptor negativity (Adjuvant Chemotherapy Analysis Cohort).

|  |  |  | **Adjuvant Hormone Therapy Analysis Cohort** | | | | **Adjuvant Chemotherapy Analysis Cohort** | | | |
| --- | --- | --- | --- | --- | --- | --- | --- | --- | --- | --- |
|  |  |  | **Non-low SES Institutions** | | **Low SES Institutions** | | **Non-low SES Institutions** | | **Low SES Institutions** | |
|  |  |  | ***Non-low SES patients*** | ***Low SES patients*** | ***Non-low SES patients*** | ***Low SES patients*** | ***Non-low SES patients*** | ***Low SES patients*** | ***Non-low SES patients*** | ***Low SES patients*** |
| **n** |  |  | 483,063 | 141,762 | 23,674 | 32,344 | 69,298 | 29,510 | 4,042 | 8,049 |
| **Age** | |  | 61.9 ± 13.2 | 60.2 ± 13.3 | 61.9 ± 12.9 | 60.0 ± 13.0 | 53.3 ± 9.9 | 52.2 ± 10.1 | 53.8 ± 9.9 | 52.1 ± 10.0 |
| **Race** | | |  |  |  |  |  |  |  |  |
|  | White | | 429,266 (88.9) | 106,877 (75.4) | 19,868 (83.9) | 22,348 (69.1) | 56,362 (81.3) | 18,628 (63.1) | 2,811 (69.5) | 4,338 (53.9) |
|  | Black | | 28,200 (5.8) | 24,545 (17.3) | 3,047 (12.9) | 8,570 (26.5) | 9,035 (13.0) | 8,887 (30.1) | 1,098 (27.2) | 3,384 (42.0) |
|  | Other | | 21,109 (4.4) | 8,967 (6.3) | 643 (2.7) | 1,233 (3.8) | 3,137 (4.5) | 1,712 (5.8) | 121 (3.0) | 269 (3.3) |
|  | Unknown | | 4,488 (0.9) | 1,373 (1.0) | 116 (0.5) | 193 (0.6) | 764 (1.1) | 283 (1.0) | 12 (0.3) | 58 (0.7) |
| **Insurance status** | | |  |  |  |  |  |  |  |  |
|  | Not insured | | 0 (0.0) | 7,175 (5.1) | 0 (0.0) | 3,751 (11.6) | 0 (0.0) | 2,120 (7.2) | 0 (0.0) | 1,251 (15.5) |
|  | Private insurance | | 276,298 (57.2) | 52,190 (36.8) | 12,431 (52.5) | 9,987 (30.9) | 55,528 (80.1) | 13,897 (47.1) | 2,913 (72.1) | 3,043 (37.8) |
|  | Medicaid | | 0 (0.0) | 32,030 (22.6) | 0 (0.0) | 6,166 (19.1) | 0 (0.0) | 8,749 (29.6) | 0 (0.0) | 2,148 (26.7) |
|  | Medicare | | 196,108 (40.6) | 47,708 (33.7) | 10,184 (43.0) | 11,354 (35.1) | 11,821 (17.1) | 4,201 (14.2) | 886 (21.9) | 1,241 (15.4) |
|  | Other Government | | 4,667 (1.0) | 1,055 (0.7) | 548 (2.3) | 262 (0.8) | 931 (1.3) | 231 (0.8) | 123 (3.0) | 80 (1.0) |
|  | Unknown | | 5,990 (1.2) | 1,604 (1.1) | 511 (2.2) | 824 (2.5) | 1,018 (1.5) | 312 (1.1) | 120 (3.0) | 286 (3.6) |
| **Percent No High School Degree** | | |  |  |  |  |  |  |  |  |
|  | 17.6% or more | | 0 (0.0) | 83,864 (59.2) | 0 (0.0) | 22,796 (70.5) | 0 (0.0) | 16,817 (57.0) | 0 (0.0) | 5,550 (69.0) |
|  | 10.9% - 17.5% | | 98,004 (20.3) | 32,129 (22.7) | 8,381 (35.4) | 6,469 (20.0) | 15,904 (23.0) | 7,166 (24.3) | 1,508 (37.3) | 1,672 (20.8) |
|  | 6.3% - 10.8% | | 154,012 (31.9) | 14,390 (10.2) | 6,794 (28.7) | 1,642 (5.1) | 22,934 (33.1) | 3,051 (10.3) | 1,204 (29.8) | 449 (5.6) |
|  | Less than 6.3% | | 167,779 (34.7) | 6,996 (4.9) | 4,972 (21.0) | 557 (1.7) | 22,284 (32.2) | 1,388 (4.7) | 752 (18.6) | 144 (1.8) |
|  | Unknown | | 63,268 (13.1) | 4,383 (3.1) | 3,527 (14.9) | 880 (2.7) | 8,176 (11.8) | 1,088 (3.7) | 578 (14.3) | 234 (2.9) |
| **Median Income Quartiles** | | |  |  |  |  |  |  |  |  |
|  | Less than $40,227 | | 0 (0.0) | 69,074 (48.7) | 0 (0.0) | 22,122 (68.4) | 0 (0.0) | 14,326 (48.5) | 0 (0.0) | 5,433 (67.5) |
|  | $40,227 - $50,353 | | 78,620 (16.3) | 30,056 (21.2) | 7,561 (31.9) | 5,936 (18.4) | 12,124 (17.5) | 6,184 (21.0) | 1,281 (31.7) | 1,448 (18.0) |
|  | $50,354 - $63,332 | | 110,313 (22.8) | 22,980 (16.2) | 6,881 (29.1) | 2,269 (7.0) | 16,356 (23.6) | 4,768 (16.2) | 1,278 (31.6) | 633 (7.9) |
|  | $63,333 or more | | 230,284 (47.7) | 15,001 (10.6) | 5,656 (23.9) | 1,015 (3.1) | 32,561 (47.0) | 3,101 (10.5) | 895 (22.1) | 278 (3.5) |
|  | Unknown | | 63,846 (13.2) | 4,651 (3.3) | 3,576 (15.1) | 1,002 (3.1) | 8,257 (11.9) | 1,131 (3.8) | 588 (14.5) | 257 (3.2) |
| **Institution Type** | | |  |  |  |  |  |  |  |  |
|  | Community Cancer Center | | 32,279 (6.7) | 10,718 (7.6) | 1,435 (6.1) | 4,202 (13.0) | 4,370 (6.3) | 2,010 (6.8) | 168 (4.2) | 849 (10.5) |
|  | Comprehensive Community Cancer Program | | 199,725 (41.3) | 59,686 (42.1) | 7,731 (32.7) | 9,815 (30.3) | 26,605 (38.4) | 10,910 (37.0) | 1,239 (30.7) | 2,147 (26.7) |
|  | Academic/Research Program | | 120,902 (25.0) | 35,986 (25.4) | 11,192 (47.3) | 14,328 (44.3) | 16,459 (23.8) | 7,621 (25.8) | 1,827 (45.2) | 3,578 (44.5) |
|  | Integrated Network Cancer Program | | 110,581 (22.9) | 27,503 (19.4) | 2,328 (9.8) | 2,224 (6.9) | 14,685 (21.2) | 5,350 (18.1) | 419 (10.4) | 513 (6.4) |
|  | Unknown | | 19,576 (4.1) | 7,869 (5.6) | 988 (4.2) | 1,775 (5.5) | 7,179 (10.4) | 3,619 (12.3) | 389 (9.6) | 962 (12.0) |
| **Charlson/Deyo Score** | | |  |  |  |  |  |  |  |  |
|  | 0 |  | 408,571 (84.6) | 115,587 (81.5) | 19,727 (83.3) | 26,080 (80.6) | 60,974 (88.0) | 24,731 (83.8) | 3,493 (86.4) | 6,746 (83.8) |
|  | 1 |  | 55,871 (11.6) | 19,342 (13.6) | 2,965 (12.5) | 4,668 (14.4) | 6,668 (9.6) | 3,724 (12.6) | 449 (11.1) | 1,019 (12.7) |
|  | 2 |  | 12,390 (2.6) | 4,566 (3.2) | 644 (2.7) | 1,056 (3.3) | 1,176 (1.7) | 707 (2.4) | 58 (1.4) | 178 (2.2) |
|  | ≥3 | | 6,231 (1.3) | 2,267 (1.6) | 338 (1.4) | 540 (1.7) | 480 (0.7) | 348 (1.2) | 42 (1.0) | 106 (1.3) |
| **AJCC Pathologic Stage** | | |  |  |  |  |  |  |  |  |
|  | 1 |  | 221,191 (45.8) | 57,182 (40.3) | 10,505 (44.4) | 12,113 (37.5) | 21,706 (31.3) | 7,364 (25.0) | 1,165 (28.8) | 1,861 (23.1) |
|  | 2 |  | 191,966 (39.7) | 60,182 (42.5) | 9,557 (40.4) | 14,057 (43.5) | 34,833 (50.3) | 15,576 (52.8) | 2,080 (51.5) | 4,251 (52.8) |
|  | 3 |  | 56,759 (11.7) | 21,097 (14.9) | 3,044 (12.9) | 5,604 (17.3) | 11,729 (16.9) | 6,181 (20.9) | 743 (18.4) | 1,850 (23.0) |
|  | Unknown | | 13,147 (2.7) | 3,301 (2.3) | 568 (2.4) | 570 (1.8) | 1,030 (1.5) | 389 (1.3) | 54 (1.3) | 87 (1.1) |
| **HER2 subtype** | | |  |  |  |  |  |  |  |  |
|  | HER2- | | 218,478 (45.2) | 61,604 (43.5) | 11,527 (48.7) | 13,838 (42.8) | 17,833 (25.7) | 7,926 (26.9) | 1,344 (33.3) | 2,282 (28.4) |
|  | HER2+ | | 22,518 (4.7) | 7,295 (5.1) | 1,356 (5.7) | 1,893 (5.9) | 4,730 (6.8) | 2,089 (7.1) | 316 (7.8) | 561 (7.0) |
|  | Unknown | | 242,067 (50.1) | 72,863 (51.4) | 10,791 (45.6) | 16,613 (51.4) | 46,735 (67.4) | 19,495 (66.1) | 2,382 (58.9) | 5,206 (64.7) |
| **Tumor Grade** | | |  |  |  |  |  |  |  |  |
|  | Well-differentiated | | 104,221 (21.6) | 27,804 (19.6) | 4,812 (20.3) | 5,924 (18.3) | 1,089 (1.6) | 378 (1.3) | 61 (1.5) | 133 (1.7) |
|  | Moderately-differentiated | | 245,578 (50.8) | 69,508 (49.0) | 11,647 (49.2) | 15,230 (47.1) | 10,148 (14.6) | 4,045 (13.7) | 605 (15.0) | 1,144 (14.2) |
|  | Poorly differentiated | | 115,505 (23.9) | 38,661 (27.3) | 6,144 (26.0) | 9,502 (29.4) | 55,521 (80.1) | 23,930 (81.1) | 3,202 (79.2) | 6,367 (79.1) |
|  | Unknown | | 17,759 (3.7) | 5,789 (4.1) | 1,071 (4.5) | 1,688 (5.2) | 2,540 (3.7) | 1,157 (3.9) | 174 (4.3) | 405 (5.0) |
| **Tumor stage** | | |  |  |  |  |  |  |  |  |
|  | 0 |  | 1,327 (0.3) | 437 (0.3) | 95 (0.4) | 121 (0.4) | 831 (1.2) | 343 (1.2) | 69 (1.7) | 98 (1.2) |
|  | 1a/b | | 19,072 (3.9) | 5,596 (3.9) | 1,013 (4.3) | 1,386 (4.3) | 3,577 (5.2) | 1,330 (4.5) | 221 (5.5) | 369 (4.6) |
|  | 1c | | 279,949 (58.0) | 74,142 (52.3) | 13,344 (56.4) | 15,868 (49.1) | 28,820 (41.6) | 10,160 (34.4) | 1,563 (38.7) | 2,560 (31.8) |
|  | 2 |  | 141,182 (29.2) | 46,514 (32.8) | 7,130 (30.1) | 11,096 (34.3) | 27,546 (39.8) | 12,744 (43.2) | 1,681 (41.6) | 3,538 (44.0) |
|  | 3 |  | 22,268 (4.6) | 7,763 (5.5) | 1,095 (4.6) | 1,894 (5.9) | 3,900 (5.6) | 2,350 (8.0) | 251 (6.2) | 754 (9.4) |
|  | 4 |  | 6,683 (1.4) | 2,729 (1.9) | 376 (1.6) | 776 (2.4) | 1,609 (2.3) | 1,050 (3.6) | 104 (2.6) | 345 (4.3) |
|  | Unknown | | 12,582 (2.6) | 4,581 (3.2) | 621 (2.6) | 1,203 (3.7) | 3,015 (4.4) | 1,533 (5.2) | 153 (3.8) | 385 (4.8) |
| **Nodal stage** | | |  |  |  |  |  |  |  |  |
|  | 0 |  | 286,620 (59.3) | 77,969 (55.0) | 13,708 (57.9) | 17,267 (53.4) | 40,145 (57.9) | 15,957 (54.1) | 2,291 (56.7) | 4,278 (53.1) |
|  | 1 |  | 68,443 (14.2) | 21,346 (15.1) | 3,516 (14.9) | 5,022 (15.5) | 8,830 (12.7) | 3,945 (13.4) | 551 (13.6) | 1,128 (14.0) |
|  | 2 |  | 30,166 (6.2) | 11,044 (7.8) | 1,608 (6.8) | 2,909 (9.0) | 5,447 (7.9) | 2,657 (9.0) | 344 (8.5) | 794 (9.9) |
|  | 3 |  | 13,750 (2.8) | 5,268 (3.7) | 802 (3.4) | 1,382 (4.3) | 2,896 (4.2) | 1,492 (5.1) | 184 (4.6) | 423 (5.3) |
|  | Unknown | | 84,084 (17.4) | 26,135 (18.4) | 4,040 (17.1) | 5,764 (17.8) | 11,980 (17.3) | 5,459 (18.5) | 672 (16.6) | 1,426 (17.7) |
| **Systemic therapy** | | |  |  |  |  |  |  |  |  |
|  | **Hormone therapy** | |  |  |  |  |  |  |  |  |
|  |  | No | 74,604 (15.4) | 25,405 (17.9) | 3,808 (16.1) | 5,989 (18.5) |  |  |  |  |
|  |  | Yes | 393,878 (81.5) | 111,305 (78.5) | 19,213 (81.2) | 25,357 (78.4) |  |  |  |  |
|  |  | Unknown | 14,581 (3.0) | 5,052 (3.6) | 653 (2.8) | 998 (3.1) |  |  |  |  |
|  | **Hormone therapy within 365 days** | | 364,539 (75.5) | 101,600 (71.7) | 18,439 (77.9) | 23,810 (73.6) |  |  |  |  |
|  | **Chemotherapy** | |  |  |  |  |  |  |  |  |
|  |  | No |  |  |  |  | 6,610 (9.5) | 3,340 (11.3) | 429 (10.6) | 889 (11.0) |
|  |  | Yes |  |  |  |  | 61,752 (89.1) | 25,784 (87.4) | 3,561 (88.1) | 7,061 (87.7) |
|  |  | Unknown |  |  |  |  | 936 (1.4) | 386 (1.3) | 52 (1.3) | 99 (1.2) |
|  | **Chemotherapy within 120 days** | |  |  |  |  | 57,381 (82.8) | 23,572 (79.9) | 3,441 (85.1) | 6,634 (82.4) |

Note: All comparisons between and within institution SES categories for each analysis cohort are statistically significant with a p<0.001.SES: socioeconomic status; AJCC: American Joint Committee on Cancer; HR: hormone receptor
